# Data on road traffic incidents for Sydney greater metropolitan area

**DOI:** 10.1016/j.dib.2023.109769

**Published:** 2023-11-04

**Authors:** V.A. Bharat Kumar Anna, Laxman Singh Bisht, Sai Chand

**Affiliations:** a Transportation Research and Injury Prevention Centre (TRIP Centre), Indian Institute of Technology Delhi, 110016, India; b Department of Transport and Planning, Faculty of Civil Engineering and Geosciences, Delft University of Technology, Stevinweg 1, Delft 2628CN, the Netherlands

**Keywords:** RTI duration, Road safety, Traffic congestion, Traffic management

## Abstract

A road network aims to facilitate the movement of commuters and goods in a safe, economical, and efficient way that contributes to growth in the economy. Road traffic incidents (RTIs), such as crashes, vehicle breakdowns, hazards, etc., are unexpected events that cause severe traffic congestion, unreliability, and pollution. The existing open-source RTI databases provide information on only a single type of incident, i.e., crashes that too focusing on the fatal ones. Other incidents, such as vehicle breakdowns, are underreported to the transport authorities as they are less severe than road traffic crashes. However, traffic congestion induced by on-road breakdowns is non-trivial, as reported by past studies. Furthermore, the existing RTI databases lack information on incident duration, a variable that indicates the time it takes for the authorities to clear the incident site and bring traffic operations back to normalcy. The increase in duration may reflect either the severity of the incident or/and the delay in emergency services and thus becomes a key indicator for traffic and safety management. Therefore, this paper aims to present the RTI data of the Sydney Greater Metropolitan Area (GMA), Australia, which includes crashes and breakdowns, along with their duration, covering 5.5 years, starting from the 1^st^ January 2017. The uniqueness of this data is that the RTI duration, i.e., the clearance time of every incident, is provided along with other details, such as vehicles involved, traffic conditions, advisories imposed, etc., over a larger area. Further, the secondary data corresponding to the road network, zonal information, socioeconomic attributes, and travel characteristics collected from various sources were also included. The curated data could be employed to examine the factors influencing RTIs at the micro (individual incident) and macroscopic (zonal) levels.

Specifications Table

**Table:** 

Subject area	Transportation engineering
More specific subject area	Transportation safety and sustainability, traffic congestion, road safety
Type of data	Tables Figures Continues and discrete data Date and time Strings and categorical data
How data was acquired	• RTI data was collected from the Open Data Hub, Transport for New South Wales (TfNSW), Australia using a Python script-based API [1]. • Road network data was obtained from OpenStreetMap using OSMnx and Rapidex [2,3]. • Bus network data was obtained from the Open Data Hub, General Transit Feed Specification (GTFS), NSW [4]. • Zonal boundaries, socio-economic attributes, and travel characteristics corresponding to Statistical Area Level-2 (SA2) were obtained from the Australian Bureau of Statistics [5].
Data format	Raw and curated data in Excel files (.xlsx and .csv), spatial data files (ESRI shapefile), and API code in Python script
Description of data collection	RTI data includes three types of incidents: crashes, vehicle breakdowns, and others. The dataset also includes the location of the incident, start and end times, date of the incident, vehicles involved, incident severity, attending groups, advice imposed, travel direction affected, traffic status, etc. The road network characteristics include road type, road length, number of lanes, number of nodes and edges, road entropy, node density, number of Cul-de-sacs, etc. The land use attributes include land use entropy and the proportion of zonal area under a specific land use category. The socio-economic attributes include population by age, family income, vehicle ownership, etc. Travel characteristics include the type of work, travel mode for making trips, etc. The location of bus stops and bus stop density from the public transit characteristics were also included in the dataset.
Data source location	City: Sydney Greater Metropolitan Area (GMA) Country: Australia
Data accessibility	Repository name: Mendeley Data Direct URL to the data: https://data.mendeley.com/datasets/cgnx2cs665
Related Research Article	Chand, S., Yee, E., Alsultan, A., & Dixit, V. V. (2021). A descriptive analysis on the impact of COVID-19 lockdowns on road traffic incidents in Sydney, Australia. *International Journal of Environmental Research and Public Health*, 18(21), 11,701. https://doi.org/10.3390/ijerph182111701

## 1 Values of the Data

• The dataset includes 85,611 incidents that took place in the entire Sydney GMA, New South Wales, Australia from 1^st^ January 2017 to 31^st^ July 2022.
• Using this dataset, crash, and breakdown frequency analysis can be done for the entire Sydney GMA.
• Considering the incident duration variable in the given dataset, researchers can analyse road network resilience to crashes and breakdowns for the entire Sydney GMA.
• Since the dataset includes the RTIs corresponding before, during, and after the COVID-19 pandemic, it would be interesting to analyse and compare incident patterns.
• With the availability of zonal information, the RTIs can be linked with the zonal information such that the dataset provides an avenue for the researchers, practitioners, and engineers to retrospect the incidents at both micro and macroscopic levels for making better incident and traffic management strategies and promote sustainable transportation.
• Overall, this dataset is unique and valuable in that it contains the duration of each incident for a long period (5.5 years), which is missing in any other publicly available datasets.

## 2 Objective

Road traffic incidents (RTIs) such as crashes, vehicle breakdowns, hazards, etc., could block the roads for a certain duration leading to congestion, unreliability in travel times, increased fuel consumption, and pollution [6], [7], [8]. Among various incidents, vehicle breakdowns are the second most happening incidents after crashes in New South Wales, Australia, and the United Kingdom [7,^9^,10]. Further, the duration of an incident is an important variable that can be used to understand the factors influencing the location or the link to recover back to its original traffic state [11]. However, the existing open-source databases provide information on only a single type of incident, i.e. crash [12,13]. Moreover, these databases lack information on the duration of crashes, and the data availability of other incident types affecting the traffic is scarce. Thus, the objective of this dataset is to provide duration along with other details of various incidents (crashes, vehicle breakdowns, and others) corresponding to Sydney GMA, Australia. Additionally, this article provided the secondary dataset that includes road network characteristics, bus transit details, travel characteristics, socio-economic attributes, etc., so researchers, practitioners, engineers, etc., may use the dataset directly for the analysis.

## 3 Data Description

**Fig. 1:**
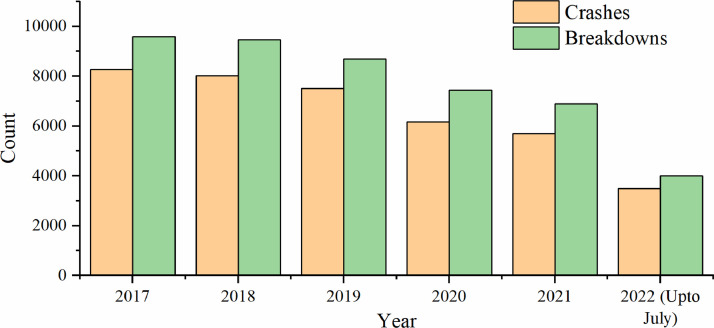
Yearly crashes and breakdowns.

**Fig. 2:**
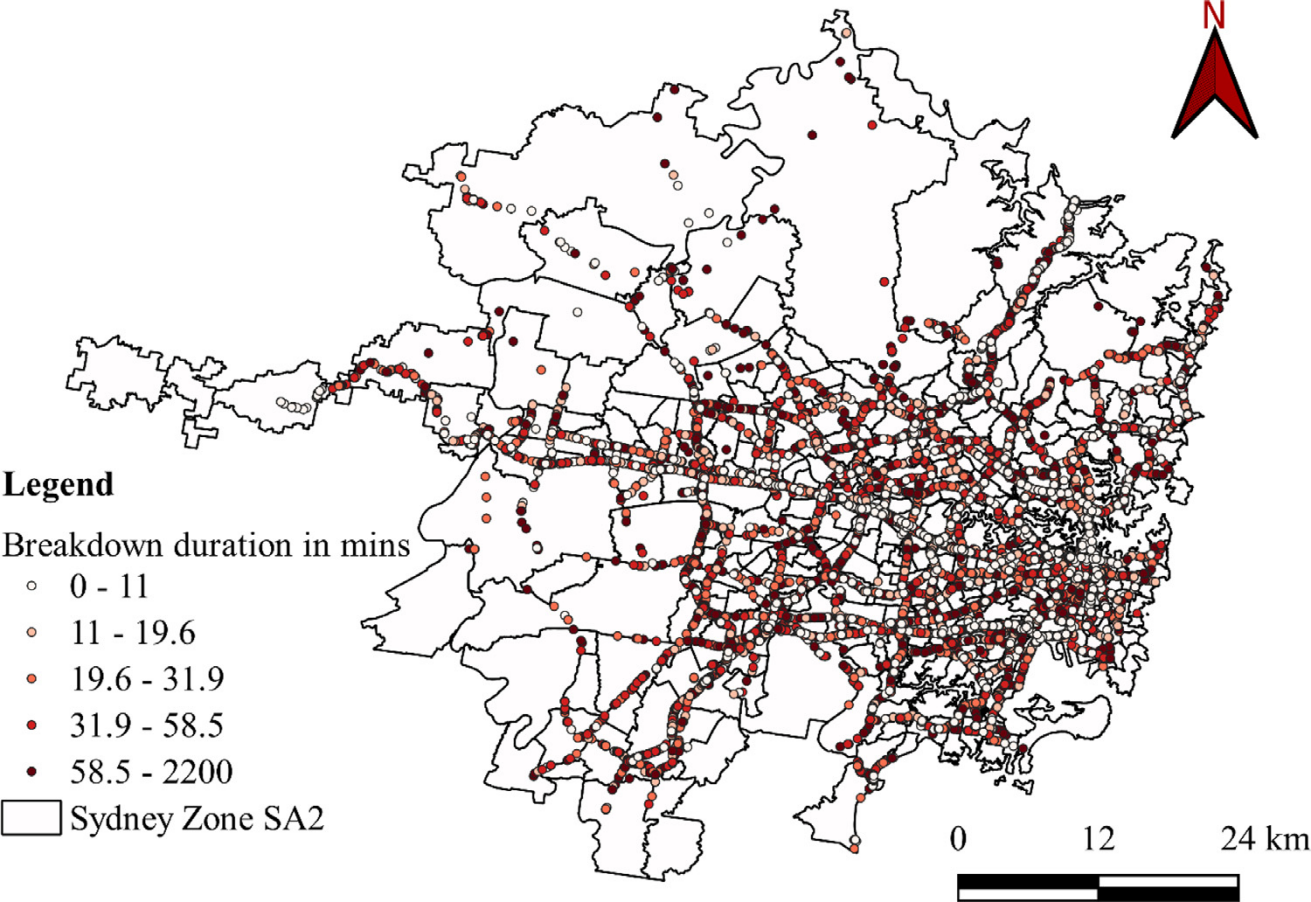
Spatial distribution of vehicle breakdowns and their durations at a microscopic level in the Sydney GMA.

**Fig. 3:**
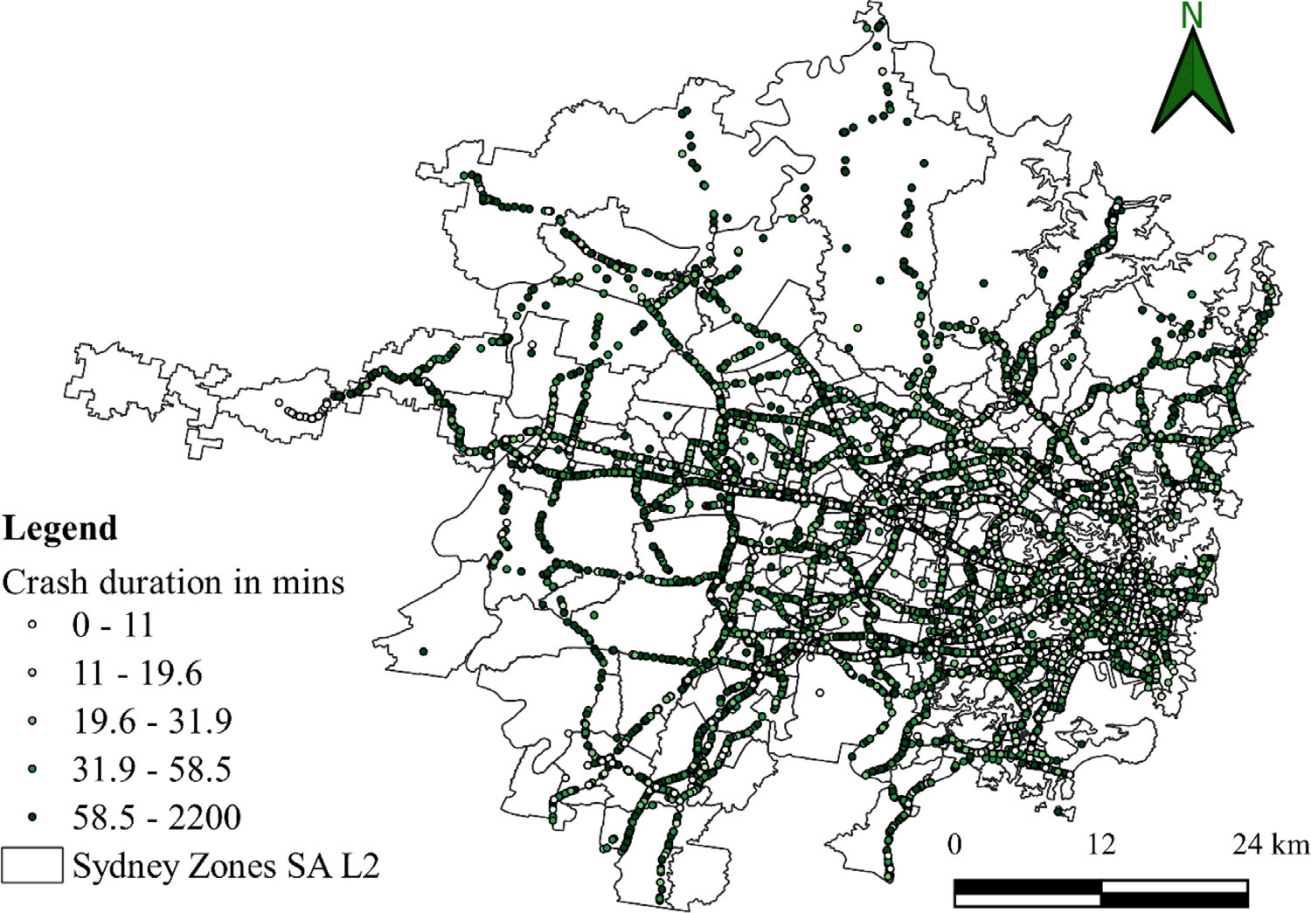
Spatial distribution of crashes and their durations at a microscopic level in the Sydney GMA.

**Fig. 4:**
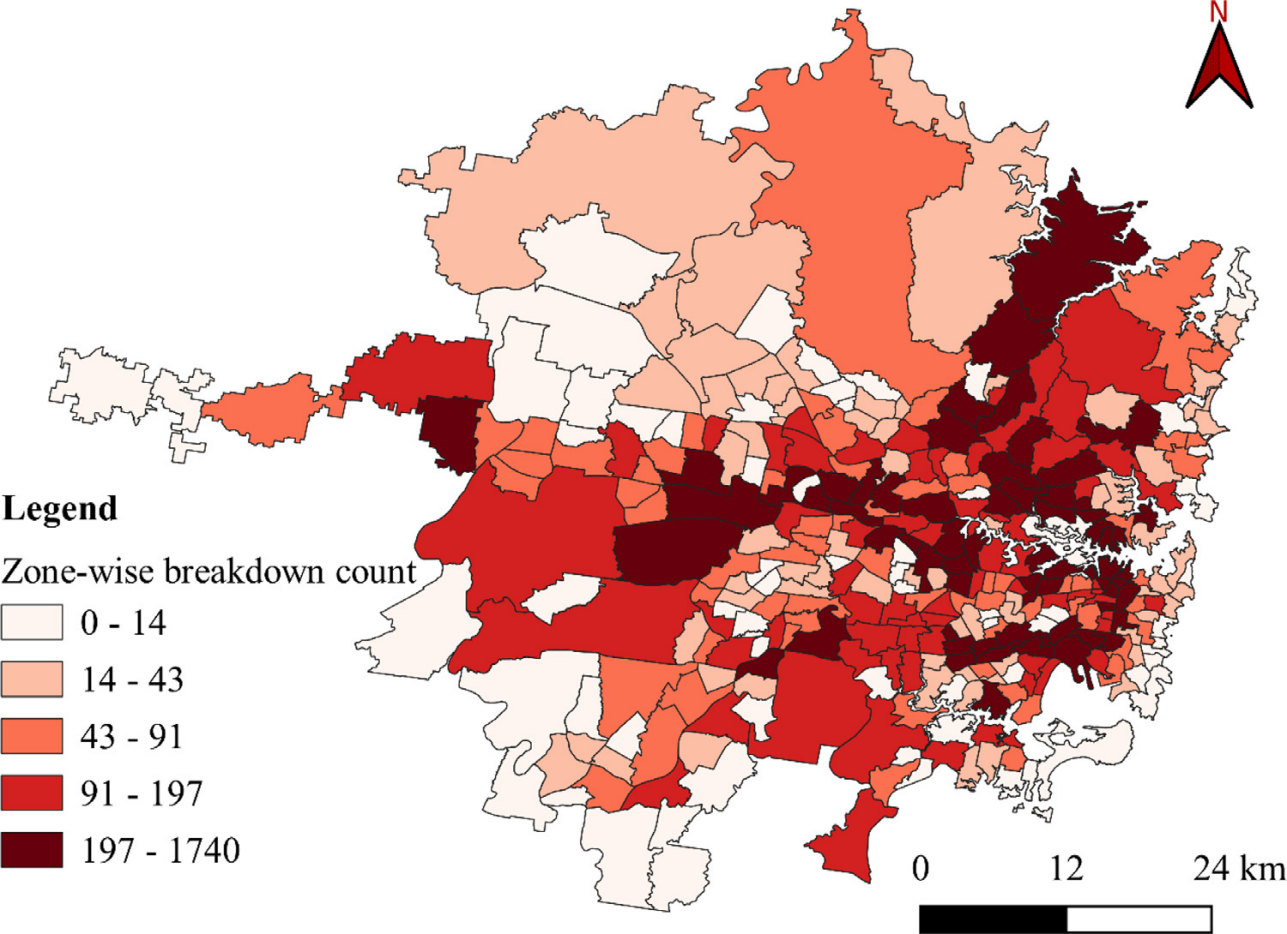
Spatial distribution of vehicle breakdowns at the macroscopic level in the Sydney GMA.

**Fig. 5:**
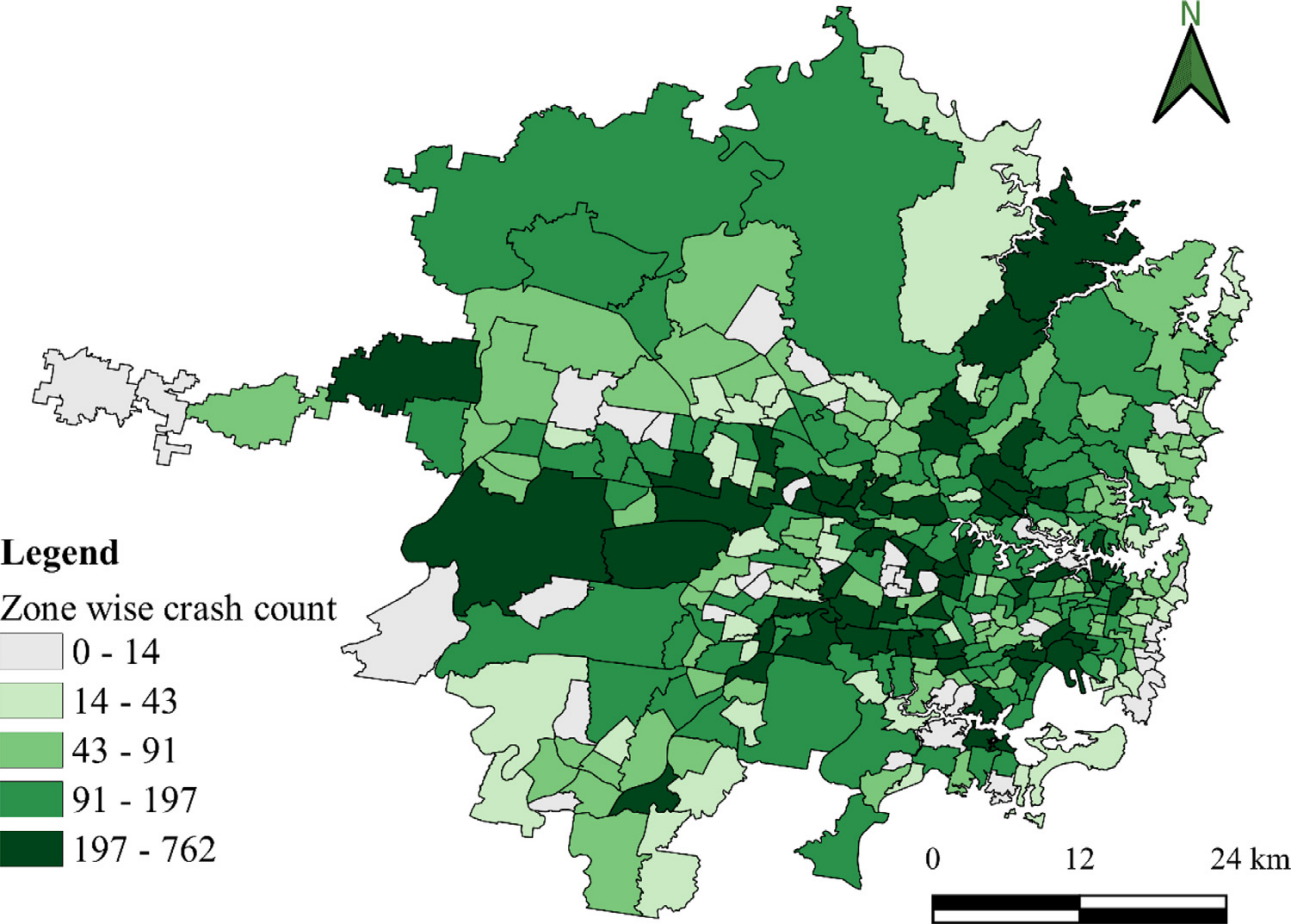
Spatial distribution of crashes at the macroscopic level in the Sydney GMA.

The datasets presented in this article are in two forms, one is the primary dataset, and the other is the secondary dataset. The primary dataset is the RTI data corresponding to the Sydney GMA of New South Wales, Australia, from 1^st^ January 2017 to 31^st^ July 2022. Both raw and curated RTIs were presented in the repository. RTI dataset comprises three types of incidents, i.e., crash, breakdown, and others. Among the three incidents, most of the incidents were crashes and breakdowns. Fig. 1 presents the yearly crash and breakdown counts, where a drop is seen for 2020 and 2021 because of COVID-19 travel restrictions. Figs. 2 and 3 present the spatial distribution of breakdowns and crashes and their durations at a microscopic level in Sydney GMA, respectively. Figs. 4 and 5 present the spatial distribution of breakdowns and crashes at the macroscopic level in Sydney GMA, respectively. The duration of RTI highlights the incident severity, i.e., high severity of the incidents would take more time to perform rescue operations, supply emergency services, and clear the location. Further, the high severity also indicates the prolonged hospitalization of the victims. Thus, as the duration of the incident increases, there is an increase in the severity of that incident. Besides, the increase in RTI duration also depends on the location of the incident and the distance to the emergency facilities (hospitals, towing vehicles, fire stations, etc.) that play a crucial role in clearing the roadways. For instance, the zones in the city outskirts are most likely covered with either parkland or empty lands with less population and infrastructure, and far from emergency facilities. This could result in an increase in the duration of the incidents even if the actual severity is less. The spatial distribution of the RTIs highlights the locations or zones that are severely affected by the incidents. The more the number of incidents in that location/zone indicates a more likelihood of occurring RTIs. The authors have no access to the categories of incident severity, in terms of injuries. The description of variables explaining the incidents is presented in metadata.

The secondary dataset comprises 49 variables curated from road network characteristics, bus transit details, land use characteristics, zonal information, socioeconomic, and travel characteristics. Metadata that explains the variables in the curated secondary dataset is also presented in the repository. Additionally, shapefiles corresponding to zonal information, RTIs, road network characteristics, bus transit details, land use characteristics, zonal information, and socioeconomic and travel characteristics were presented in the repository. An API code that is used to retrieve the RTIs from the Open Data Hub is also presented in the repository. Interested users may use the code by simply replacing their API key to retrieve the data. All the datasets and code in various formats were uploaded to the Mendeley data repository [14]. Table 1 briefly describes all the files available in the Mendeley data repository.

**Table 1:** List and a short description of the files in the Mendeley data repository.

S. No	Data file name	Short description of the file	Format/s
1	Road Traffic Incident Dataset-Raw	This raw road traffic incident dataset was downloaded using a Python script-based API from the Open Data Hub [1].	.csv
2	Final Road Traffic Incidents	The file is the processed road traffic incident dataset.	.xlsx
3	Metadata for final road traffic incidents	This file presents a description of the variables in the ‘Final Road Traffic Incidents’ file.	.xlsx
4	Sydney Zones SA2 variables_4	The file includes the final 49 variables corresponding to the road network, bus transit, land use, socioeconomic and travel characteristics for SA2.	.xlsx
5	Metadata for Sydney Zones SA2 variables_4	This file presents a description of the variables in the ‘Sydney Zones SA2 variables_4′ file.	.xlsx
6	API code for RTI Data	Python script-based API code for downloading the incident data from the Open Data Hub	.py
7	Breakdowns at Sydney zones SA2	It is a shape file. This file links breakdown incidents to Sydney GMA zones at SA2.	.zip
8	Crashes at Sydney zones SA2	It is a shape file. This file links crash incidents to Sydney GMA zones at SA2.	.zip
9	Incidents at Sydney zones SA2	It is a shape file. This file links all incidents to Sydney GMA zones at SA2.	.zip
10	Sydney zones SA2 breakdown count	It is a shape file. The file contains the breakdown count for Sydney GMA zones at SA2.	.zip
11	Sydney zones SA2 crash count	It is a shape file. The file contains the crash count for Sydney GMA zones at SA2.	.zip
12	Sydney zones SA2 incident counts	It is a shape file. The file contains the incident count (all incidents) for Sydney GMA zones at SA2.	.zip
13	Sydney Zones SA2 Edges	It is a shape file. This file links the edges to Sydney GMA zones at SA2.	.zip
14	Sydney Zones SA2 Mesh Blocks	It is a shape file. The mesh blocks are linked to Sydney GMA zones at SA2 in this file.	.zip
15	Sydney Zones SA2 Nodes	It is a shape file. In this file, the nodes are linked to Sydney GMA zones at SA2.	.zip
16	Sydney zones SA2	It is a shape file. This file presents the details of the Sydney GMA zones for SA2	.zip

## 4 Experimental Design, Materials, and Methods

This section describes the methods or approaches that were used to acquire the primary and secondary datasets from various sources.

### 4.1 Road traffic incident data

RTIs are unexpected events that cause disruption to the traffic flow. The disruption could range from a few minutes to hours, depending on the severity of the incident. In this data paper, the authors present the RTIs corresponding to crashes, vehicle breakdowns, and others for the Sydney GMA of New South Wales, Australia. In New South Wales, the incidents were detected through CCTV-based automatic incident detection, the facility's operator, and other manual entries. All the incidents were recorded in Open Data Hub, a central open data repository for all Transport for New South Wales (TfNSW), established in 2016. Some of the incidents were found to have a duration of less than a minute. A possible reason could be due to non-CCTV-based incident records, where the incidents might be detected many minutes after the start of the incident and/or were resolved by the same person who reported it. This reason was mentioned in Moylan et al. [11], where the authors used a similar dataset for Sydney. Thus, the RTI data was retrieved from the Open Data Hub through an application programming interface (API) query using Python script [1]. In a single API query, the researchers can collect the data for only 90 days; thus, multiple queries were requested to obtain the incident data from 1^st^ January 2017 to 31^st^ July 2022. Each incident is defined by an ID, category of the incident, geo-location (longitude and latitude) that includes travel direction, street, and suburb name, start and end times, vehicles involved, vehicle type, attending groups, advisories, traffic volumes, etc. Unfortunately, the information about the drivers and the victims involved in the incident is not available.

In the original/raw dataset, each RTI record has multiple entries at respective times, where the first entry is about reporting the start of the incident, and the last entry is about the closing remarks of the RTI (when the traffic operations became normal). In between, multiple entries were created for updating about the status of the RTI at respective times, such as advisories issued, measures taken to clear the road where the incident took place, etc. In this dataset, we considered the incident duration as the time between the first and last available records for an incident. In the raw dataset, there were several redundant and duplicate RTIs. The unique and valid RTIs were identified based on the first and last entries and also based on the change in the advisory. For each incident, there were two advisories. In the first advisory, i.e., advice_A, a message was displayed to the road users about the incident. In the second advisory, i.e., advice_B, another message was displayed to the road users about the action taken by either the police or the road maintenance agency. Thus, after removing the redundant and duplicate RTIs, there were 85,611 unique RTIs during this period, of which 39,165 were crashes and 46,085 were breakdowns that were spread across 333 zones within the Sydney GMA.

### 4.2 Road network data

The road network metrics, such as length of the road, number of edges, number of nodes, number of lanes corresponding to each road category, etc., were extracted from OpenStreetMap using OSMnx and Rapidex in Geo Package format [2,3]. The OSMnx and Rapidex-based road network has several advantages over the road network imported directly from Open Street Map (OSM) and other network sources. One of the main advantages is that they can correct and simplify the network topology into multidigraphs. The road network obtained from OSMnx and Rapidex would be in the form of edges and nodes where all the edges are connected through nodes. The OSM defines the edges in the road network into eight categories based on the functional road hierarchy. The eight categories are motorways, trunk roads, primary roads, secondary roads, tertiary roads, residential roads, living streets, and unclassified roads. The nodes in the network are classified into four categories based on the number of edges connected to a particular node, such as the number of cul-de-sacs, nodes with two edges, three edges, and four edges. Besides, the GTFS public transit data, such as bus stops with locations in the text format, were directly downloaded from the Open Data Hub [4]. Apart from the above data, the heterogeneity of the road network in each zone was estimated through entropy using Eq. (1).

(1) $$H\left(X_{j}\right)=\;-\sum_{i=1}^{I}p_{i}\;log_{2}\left(p_{i}\right)$$

Where *H*(*X_j_*) is the entropy of roads in *j*^th^ zone; *I* is the number of edge categories in a road network within a zone; $p_{i}$ is the proportion of each edge category *i* in the road network within the zone. A few parameters that describe the network connectivity, meshedness coefficient index (α), and completeness index (ρ) were estimated using Eqs. (2) and 3. The average number of edges connected to each node was estimated as the average node degree.

(2) $$\alpha_{j}=\frac{m_{j}-n_{j}+1}{2n_{j}-5}$$

(3) $$\rho_{j}=\frac{n_{j}}{\left(m_{j}^{2}-m_{j}\right)}$$

Where α is the meshedness coefficient index for zone *j*; ρ is the completeness index for zone *j*; m is the number of edges in the network for zone *j*; n is the number of nodes in the network for zone *j*.

### 4.3 Land use characteristics, socioeconomic and travel characteristics

The land use characteristics, socio-economic attributes, and travel characteristics for the entire Sydney GMA at SA2 were obtained from the Australian Bureau of Statistics [5]. Here, the SA2 is the zonal area representing the census boundaries. According to the SA2, the entire Sydney GMA was divided into 333 zones. Socio-economic attributes and travel characteristics include population by age, sex, household income, personal income, occupation, vehicle ownership, mode of travel to work, etc. However, the data in its current form are not readily usable for analysis purposes and they were curated to a few variables that are useful for the analysis. The original land use is characterized in the form of mesh blocks, the smallest geographic unit for which statistical data is collected and processed in Australia [15]. The mesh blocks are aggregated to get the percentage of land used for different purposes, such as commercial, hospital, residential, education, industrial, parkland, etc., for SA2. The entropy of land use was measured using Eq. (1) at SA2. All the data in the form of GIS shape files and Excel formats were directly downloaded from the Australian Bureau of Statistics.

The final land use, socioeconomic, and travel characteristics that were included in the dataset are as follows.

1. Land use characteristics include the area of land used for a specific purpose and the entropy of land use.
2. The socioeconomic characteristics that include total population, the average number of persons in a family household, the percentage of white- and blue-collar job holders, the percentage of dwellings with zero, 1, 2, 3, and 4 or more vehicle ownership, the percentage of unemployment, average monthly income.
3. The travel characteristics include travel to work by means of various transport modes such as public transport (bus, train, tram, ferry), taxi/rideshare, personal car, car as a passenger, and other modes.

Thus, altogether, the dataset comprises 49 variables, and all these variables were extracted for SA2 level. All the data presented in this paper was retrieved from the Open Data Hub and Australian Bureau of Statistics that can be redistributed and reused under a Creative Commons Attribution 4.0 International License [16]. All the datasets and code in various formats were uploaded to the Mendeley data repository [14], and a URL link to download is also provided in this paper.

## Ethics Statement

The work does not involve human subjects, animal experiments, or any data collected from social media platforms.

## CRediT authorship contribution statement

**V.A. Bharat Kumar Anna:** Data curation, Writing – original draft, Writing – review & editing. **Laxman Singh Bisht:** Data curation, Writing – original draft, Writing – review & editing. **Sai Chand:** Conceptualization, Writing – original draft, Writing – review & editing, Supervision.

## Data Availability

Road Traffic Incident (RTI) Data for Sydney Greater Metropolitan Area (Reference data) (Mendeley Data)
